# Predictive Factors for Non-Sentinel Lymph Node Metastasis in the Case of Positive Sentinel Lymph Node Metastasis in Two or Fewer Nodes in Breast Cancer

**DOI:** 10.14740/jocmr2195w

**Published:** 2015-06-09

**Authors:** Chie Toshikawa, Yu Koyama, Masayuki Nagahashi, Kumiko Tatsuda, Kazuki Moro, Junko Tsuchida, Miki Hasegawa, Toshiyuki Niwano, Naoko Manba, Mayuko Ikarashi, Hitoshi Kameyama, Takashi Kobayashi, Shin-ichi Kosugi, Toshifumi Wakai

**Affiliations:** a Division of Digestive & General Surgery, Niigata University Graduate School of Medical & Dental Sciences, 1-757 Asahimachi, Niigata, Niigata 951-8510, Japan; b Department of Nursing, Niigata University Graduate School of Health Sciences, 2-746 Asahimachi, Niigata, Niigata 951-8518, Japan; c Department of Digestive and General Surgery, Uonuma Institute of Community Medicine, Niigata University Medical and Dental Hospital, 4132 Urasa, Minami-Uonuma, Niigata 949-7302, Japan

**Keywords:** Breast cancer, Sentinel lymph node, Non-sentinel lymph node, Metastasis, Lymphatic involvement, Invasive tumor size

## Abstract

**Background:**

In breast cancer, recent clinical trials have shown that sentinel lymph node biopsy (SLNB) alone without axillary lymph node dissection results in excellent prognosis if there is sentinel lymph node (SLN) metastasis in two or fewer nodes. The aim of the present study was to investigate the association between non-SLN metastasis and clinicopathological factors in case of SLN metastasis in two or fewer nodes in breast cancer.

**Methods:**

Patients who underwent SLNB for invasive breast cancer and were found to have positive SLN in two or fewer nodes were evaluated. The associations between non-SLN metastasis and clinicopahological factors were examined. Statistical analyses were performed using the Mann-Whitney and Chi-square tests, with statistical significance set at P < 0.05.

**Results:**

A total of 358 patients were enrolled during the study period and all of these patients were female and 54 patients had SLN metastasis (15%). Positive SLN in two or fewer nodes was identified in 44 patients (81.5%). Among these patients, 17 (38.6%) were found to have non-SLN metastasis. Non-SLN metastasis was associated with invasive tumor size (P = 0.015) and lymphatic involvement (P = 0.035). Multivariate analysis showed that tumor size (P = 0.011) and lymphatic involvement (P = 0.019) remained significant independent predictors of non-SLN metastasis, and that an invasive tumor size cut-off point of 28 mm was useful for dividing patients with positive SLN in two or fewer nodes into non-SLN-positive and non-SLN-negative groups.

**Conclusions:**

Non-SLN metastasis was found in more than 30% of patients with SLN metastasis present in two or fewer nodes. Large tumor size and the presence of lymphatic involvement were significantly associated with non-SLN metastasis.

## Introduction

Non-sentinel lymph node (SLN) metastasis has been an important issue for planning further treatment of node positive breast cancer, because axillary lymph node metastasis has been a strong prognostic indicator for the patients with invasive breast cancer while new tumor markers have been widely studied [1-3]. Axillary lymph node dissection (ALND) is the standard management approach for preoperatively diagnosed node-positive breast cancer [4]. Since the 1990s, the introduction of sentinel lymph node biopsy (SLNB) has resulted in changes in the management of the axilla [5]. SLNB for clinically N0 breast cancer and ALND for positive SLN have become the standard procedures.

Recent clinical trials suggest that there is no difference in outcome between patients with positive SLN if they are treated with ALND or given no further axillary surgery [6, 7]. These studies raise doubts concerning the role of SLNB. A new trial compared SLNB with the assessment of whether an axillary ultrasound is negative in patients with small breast cancer [8]. SLN metastasis is observed in approximately 30% of SLNBs [9], so it is important to predict the axillary node status before SLNB. Various clinicopathological factors have been identified as independent predictors of axillary lymph node metastasis in early stage breast cancer [10]. These factors include clinical palpability [11-14], tumor size [11-17], lymphatic or vascular involvement [11-15, 17], tumor grade [11, 14], hormone receptor (HR) status [16, 17], age [12, 15, 16], and molecular subtype classification [3, 10, 18-26]. Predicting the non-SLN status is important because both the American College of Surgeons Oncology Group (ACOSOG) Z0011 trial [6] and the International Breast Cancer Study Group Trial 23-01 (IBCSG 23-01) [7] indicated that ALND should be avoided if SLN metastases are detected in only one or two nodes. Analytical tools have been developed to predict the risk of non-SLN metastasis if positive SLNs are found [27-33], but these tools yield a false negative rate of 7-41% (ALND for < 10% risk of non-SLN metastasis) [34]. Recently, we reported that SLN metastasis was associated with younger age, large tumor size and prominent lymphovascular involvement; however, non-SLN metastasis was hard to predict using clinicopathological factors [35].

The aim of the present study was to investigate the association between non-SLN metastasis and clinicopathological factors, particularly in the case of SLN metastasis in two or fewer nodes, in breast cancer.

## Materials and Methods

### Patient selection

Patients with invasive breast cancer who underwent SLNB at Niigata University Hospital between January 2010 and December 2014 were eligible for inclusion in the study, which was a retrospective chart review. ALND was performed in patients with macro- and micrometastasis in SLN; however, ALND was avoided in patients with isolated tumor cells in SLN. Of the patients who were positive for SLN metastasis, those with SLN metastases in two or fewer nodes were evaluated further. Only those patients with complete data for clinicopathological factors (age, clinical and pathological tumor size, HR and human epidermal growth factor receptor 2 (HER2) status, and Ki-67 labeling index) were enrolled in the study (n = 44). The data for these patients were analyzed following approval from the Institutional Review Board.

### Pathological assessment

Immunohistochemical (IHC) estrogen receptor (ER) and progesterone receptor (PR) status was assessed and tumors were deemed positive for each receptor if at least 10% of the invasive tumor cells in a section exhibited nuclear staining for that particular receptor. HER2 expression was examined by IHC. A gene amplification assay using fluorescence *in situ* hybridization (FISH) was used in cases when it was difficult to decide the HER2 status by IHC. Ki-67 was also examined by IHC, and the results are expressed as the percentage of tumor cells stained by the antibody, as described previously [36]. Hematoxylin-eosin staining was used to assess lymphatic and vascular involvement, as well as histologic grading, which was defined according to the Scarff-Bloom-Richardson system [37]. SLN metastasis was assessed by intraoperative examination of frozen sections, as well as being re-evaluated postoperatively using fixed sections. Breast cancer staging was according to the TNM classification as proposed by the American Joint Committee on Cancer (AJCC). All IHC evaluations were performed by several well-trained pathologists.

Patients were assigned into four subgroups, as proposed in the St Gallen International Expert Consensus [38] based on the results of their ER, PR, and HER2 status and Ki-67 leveling index [39], as follows: 1) a luminal A group that was ER or PR positive, HER2 negative, and had a Ki-67 labeling index < 14%; 2) a luminal B group that was ER or PR positive, HER2 positive, or had a Ki-67 labeling index (≥ 14%; 3) an HER2 group that was ER and PR negative and HER2 positive; and 4) a triple negative group that was negative for ER, PR, and HER2.

### Statistical analysis

The associations between non-SLN metastasis and clinicopahological factors (age, invasive tumor size, nulcear grade, lymphatic or venous involvement, ER and/or PR status, HER2 status, molecular subtypes, and Ki-67 labeling index) were examined. Statistical analyses were performed using Mann-Whitney’s U-test and the Chi-squared test, and multivariate analysis was performed using the logistic regression model. The diagnostic accuracy of invasive tumor size was assessed by receiver operating characteristic (ROC) analysis. The area under the ROC curve (AUC) was used to measure model discrimination. The AUC can range from 0.5 (which indicates a test with no information) to 1.0 (which indicates a perfect test). Statistical significance was set at P < 0.05.

## Results

### SLN and non-SLN metastasis

A total of 358 patients were enrolled during the study period. All patients were female and 54 had SLN metastasis (15%). Of the patients who were positive for SLN metastases, positive SLN in two or fewer nodes were obseved in 44 patients (81.5%). Among these 44 patients, non-SLN metastasis was observed in 17 patients (38.6%). Thirty-four patients (72.3%) had positive SLN in one node and 10 patients (22.7%) had positive SLN in two nodes.

### Patient characteristics and clinicopathological factors

Mean patient age was 51.6 years; however, there was no association between age and non-SLN metastasis (Table 1). Univariate analysis revealed that non-SLN metastasis was significantly associated with lymphatic involvement (P = 0.035); however, there was no significant association between non-SLN metastasis and invasive tumor size, nulcear grade, venous involvement, ER and/or PR status, HER2 status, molecular subtype, or Ki-67 labeling index.

**Table 1:** Association Between Non-Sentinel Lymph Node Metastasis and Clinicopathological Features (n = 44)

Characteristics	No. of patients	Non-SLN metastasis		P value
		Negative (%)	Positive (%)	
Age (years)				> 0.999
≤ 50	25	15 (60.0)	10 (40.0)	
> 50	19	12 (63.2)	7 (36.8)	
Invasive tumor size (mm)				0.062
≤ 20	22	17 (77.3)	5 (22.7)	
> 20	22	10 (45.5)	12 (54.5)	
Histological grade				0.108
I	28	20 (71.4)	8 (28.6)	
II-III	16	7 (43.8)	9 (56.2)	
Lymphatic involvement				0.035
Negative	32	23 (71.9)	9 (28.1)	
Positive	12	4 (33.3)	8 (66.7)	
Venous involvement				> 0.999
Negative	37	23 (62.2)	14 (37.8)	
Positive	7	4 (57.1)	3 (42.9)	
ER and/or PR				> 0.999
Negative	3	2 (66.7)	1 (33.3)	
Positive	41	25 (61.0)	16 (39.0)	
HER2				> 0.999
Negative	36	22 (61.1)	14 (38.9)	
Positive	8	5 (62.5)	3 (37.5)	
Molecular subtype				0.867
Luminal A	33	20 (60.6)	13 (39.4)	
Luminal B	7	4 (57.1)	3 (42.9)	
HER2	1	1 (100)	0 (0)	
Triple negative	3	2 (66.7)	1 (33.3)	
Ki-67 labeling index				0.359
< 14%	25	17 (68.0)	8 (32.0)	
≥ 14%	19	10 (52.6)	9 (47.4)	

SLN: sentinel lymph node; ER: estrogen receptor; PR: progesterone receptor.

### Invasive tumor size and non-SL metastasis

**Figure 1:**
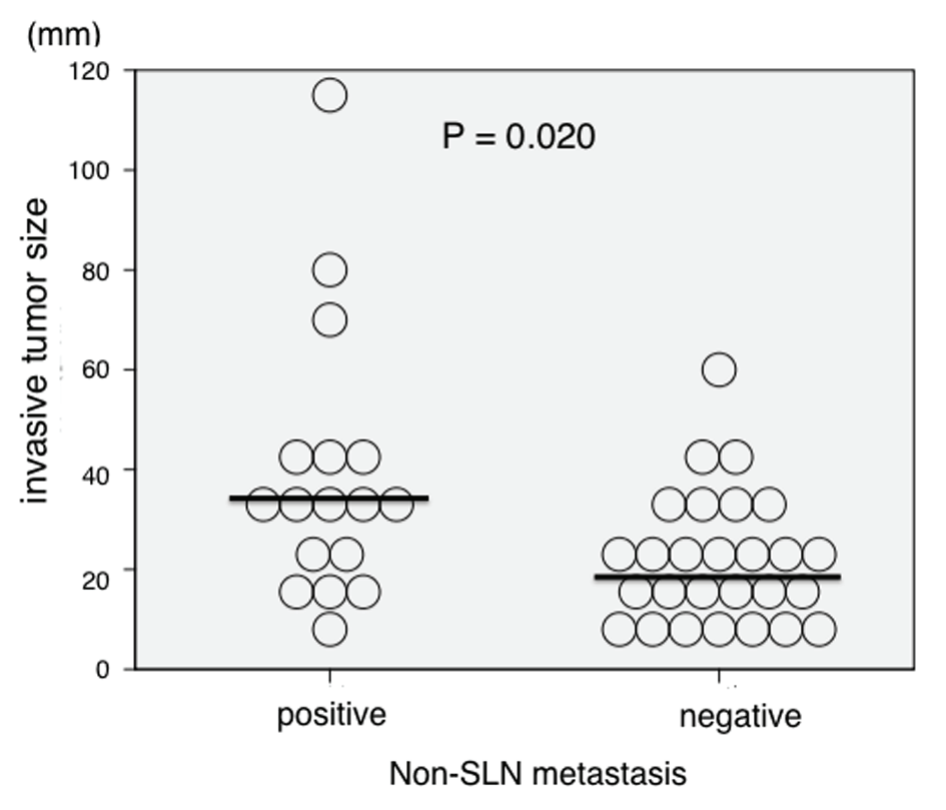
Association between invasive tumor size and non-sentinel lymph node (SLN) metastasis (n = 44). Invasive tumor size was significantly larger in patients who were positive for non-SLN metastasis compared with those who were negative (median size 35.0 vs. 20 mm, respectively; P = 0.020).

**Figure 2:**
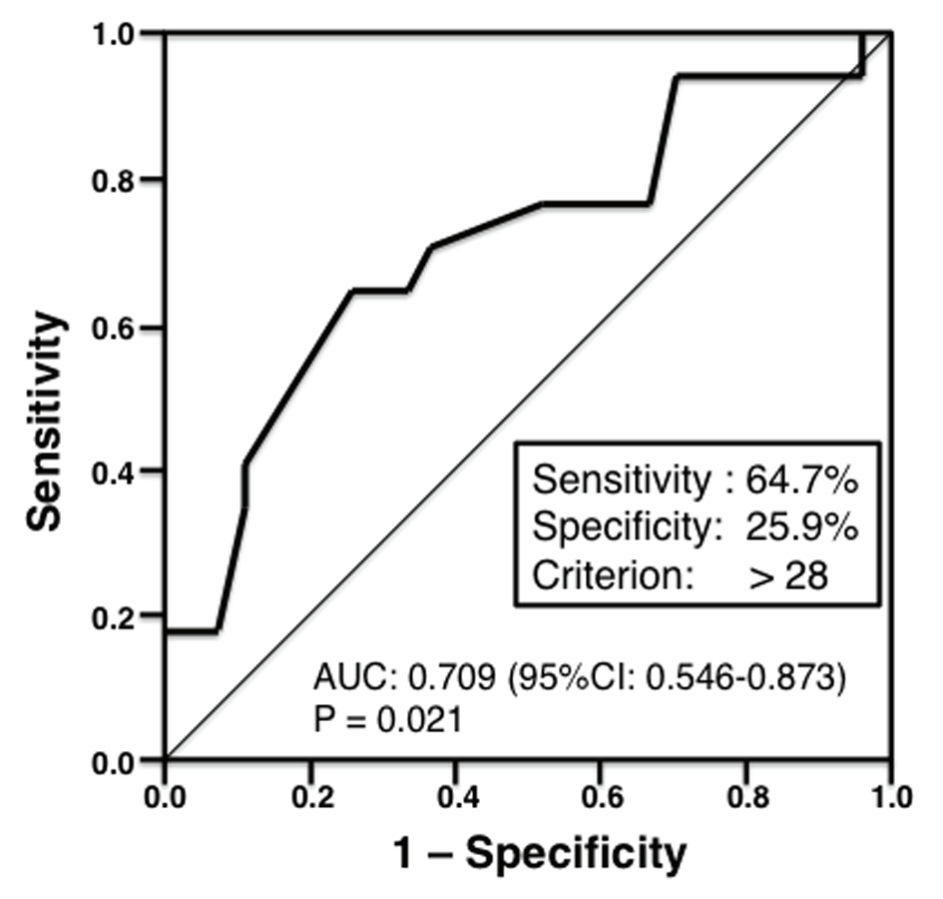
Receiver operating characteristic (ROC) analysis of invasive tumor size (n = 44). The ROC analysis identified a cut-off point of 28 mm (area under the curve 0.709; sensitivity 64.7%; specificity 25.9%; P = 0.021).

Conversely, median invasive tumor size was significantly larger in patients who were positive for non-SLN metastasis compared with metastasis negative patients (35.0 vs. 20 mm, respectively; P = 0.020; Fig. 1). However, there was no significant association between non-SLN metastasis and invasive tumor size if the cut-off point was 20 mm. Therefore, we invastigated the threshold value for invasive tumor size that differentiated patients with non-SLN metastasis using ROC analysis. ROC analysis identified a cut-off point of 28 mm (AUC 0.709; sensitivity 64.7%; specificity 25.9%; P = 0.021; Fig. 2). We then used this cut-off point of 28 mm to reassess patients. As indicated in Table 2, using a cut-off value of 28 mm resulted in a significant association between invasive tumor size and non-SLN metastasis (P = 0.015).

**Table 2:** Non-Sentinel Lymph Node Metastasis and Cut-Off Points of Invasive Tumor Size (n = 44)

Invasive tumor size	No. of patients	Non-SLN metastasis		P value
		Negative (%)	Positive (%)	
Cut-off point 20 mm				0.062
≤ 20 mm	22	17 (77.3)	5 (22.7)	
> 20 mm	22	10 (45.5)	12 (54.5)	
Cut-off point 28 mm				0.015
≤ 28 mm	26	20 (76.9)	6 (23.1)	
> 28 mm	18	7 (38.9)	11 (61.1)	

SLN: sentinel lymph node.

### Multivariate analysis of predictor for non-SLN metastasis

Multivariate analysis showed that both lymphatic involvement (P = 0.019; relative risk (RR): 7.255; 95% confidence interval (CI): 1.388 - 37.929) and invasive tumor size (P = 0.011; RR: 7.083; 95% CI: 1.560 - 32.153) were significant factors predicting non-SLN metastasis (Table 3).

**Table 3:** Multivariate Analysis of Clinicopathological Factors (n = 44)

Variable	RR (95% CI)	P value
Lymphatic involvement		
Negative	1.000	
Positive	7.255 (1.388 - 37.929)	0.019
Invasive tumor size		
≤ 28 mm	1.000	
> 28 mm	7.083 (1.560 - 32.153)	0.011

RR: relative risk; CI: confidence interval.

## Discussion

SLNB for clinically node-negative breast cancer has become a standard procedure worldwide and it is important to determine pathologically whether the node is negative before surgery. Although ALND has been the standard procedure if patients are positive for SLN metastasis, recent clinical trials suggest that ALND is unnecessary if positive SLN metastasis is detected in one or two nodes. The ACOSOG Z0011 study [6] showed that SLNB alone without ALND results in extremely low locoregional recurrence and excellent overall survival comparable to that in patients undergoing ALND if SLN metastasis is present in two or fewer nodes. In the present study, we investigated patients with SLN metastasis in two or fewer nodes to elucidate predictive clinicopathological factors in the case of SLN metastasis in two or fewer nodes. The results revealed a > 30% risk for non-SLN metastasis even if SLN metastasis occurred in one or two nodes only. Breast surgeons must understand the risk of renmant non-SLN metastasis if they do not perform additional ALND for patients with SLN metastasis in two or fewer nodes. Thus, predictions of non-SLN metastasis in the case of positive SLN metastasis in two or fewer nodes are necessary when making decisions regarding additional ALND.

Previous studies have reported that younger age, higher pT stage, or lymphovascular involvement are independent predictors of SLN metastasis [11-17], with HR and histological grade good predictors of positive SLN [11, 14, 16, 17]. With regard to the prediction of non-SLN metastases, younger age, large tumor size or higher pT stage, lymphovascular involvement, extracapsular invasion, the ratio of positive SLNs to the total number of harvested SLNs, or total tumoral load in the SLNs assessed by one-step nucleic acid amplification have been reported as useful markers [40-44]. However, these studies were not performed in patients with positive SLN metastasis in two or fewer nodes. Therefore, in the present study, we investigated the association between non-SLN metastasis and clinicopathological factors, particularly in the case of SLN metastasis in two or fewer nodes, in breast cancer. The findings show that invasive tumor size and lymphatic involvement are significantly associated with non-SLN metastasis in the case of SLN metastasis in two or fewer nodes. We investigated the association between non-SLN metastasis and invasive tumor size initially using a cut-off point of 20 mm because 20 mm is the point dividing T1 and T2; however, there was no significant association between non-SLN and invasive tumor size using 20 mm as the cut-off point. However, invasive tumor size was significantly larger in patients who were positive for non-SLN metastasis compared with those who were metastasis negative (Fig. 1). So, we invastigated the threshold value of invasive tumor size that differentiated patients with non-SLN metastasis using ROC analysis. The results of ROC analysis identified 28 mm as the most useful cut-off point to discriminate between non-SLN metastasis positive and negative patients.

The main limitations of the present study include the retrospective nature of the analysis and the small number of patients who have had positive SLN metastasis at two or fewer nodes. We believe, however, that these limitations did not greatly affect the results of the study as the differences between non-SLN positive and negative were too marked to have resulted from bias. Our results thus provide useful information on the risk factors for renmant non-SLN metastasis under the condition of SLN metastasis at two or fewer nodes.

Finally, both lymphatic involvement and invasive tumor size (cut-off point 28 mm) were independent predictors of non-SLN metastasis in the case of SLN metastasis in two or fewer nodes. Systemic therapies, including hormone therapy, cytotoxic chemotherapy, and/or molecular targeting drugs and/or radiation therapy, remain extremely important, particularly in the treatment of node-positive breast cancer. Therefore, the results of the present study will contribute to decision-making with regard to the addition of ALND, systemic therapy, and/or radiation therapy in the case of SLN metastasis in two or fewer nodes.

### Conclusion

Non-SLN metastasis was found in more than 30% of patients in the present study, even if SLN metastasis was present in two or fewer nodes, and large tumor size and the presence of lymphatic involvement were significanly associated with non-SLN metastasis.
